# Achieving the Millennium Development Goals in Bangladesh

**Published:** 2008-09

**Authors:** David A. Sack

**Affiliations:** ICDDR, B, Mohakhali, Dhaka 1212, Bangladesh

In early 2006, scientists at the International Centre for Diarrhoeal Disease Research, Bangladesh (ICDDR, B) proposed to the Office of the Population, Health, and Nutrition at the USAID Mission in Dhaka a review of some major health issues of Bangladesh—at least those in which ICDDR, B has expertise. The aim of this review was to identify selected key issues, try to understand the trends over the last one to two decade(s), and to interpret these trends in the light of Millennium Development Goals (MDGs). It was hoped these trends would show that Bangladesh was on target to meet these goals, or if it was not on a successful track, to suggest reasonable strategies to improve the trends.

Obviously, there are many important health issues outside the scope of the ICDDR, B's expertise, e.g. diabetes, heart disease, cancer, and many others. These are critical issues for the country, but these have not been identified as MDG targets, nor are these within the expertise of ICDDR, B; these are not, thus, included in this volume, although these may be extremely important for the overall provision of healthcare in the country.

ICDDR, B is organized into thematic programmes as shown in the box. The members of each programme have discussed the challenge of identifying the key issues in their thematic area and have provided the information for each of the papers in this report. Some papers submitted to me were too detailed and had to be condensed to reduce their size. However, I have attempted to include the essence of the thinking of the groups. In each case, a writing team has contributed to the paper, and a smaller group has actually drafted the version submitted.

For each of the identified issues, there were two cross-cutting and inter-related themes in the background of the discussion. These may not always be stated explicitly in the text, but these clearly form an underlying concept for all our research and evaluation at the Centre. These are issues of *equity and gender.* It is not fair that basic health services would be available to some groups and not others. It is not fair that girls have higher degrees of malnutrition than boys, that women receive treatment for serious chronic conditions later than men, or that women proceed through pregnancy and delivery without the services needed to protect their health and their life. These equity and gender concerns partly relate to society and cultural practices, but they also relate to the health system itself. Regardless of the source of the inequity or bias, the health system must understand them and adjust to meet the needs of the vulnerable groups and females.

**Programmes at ICDDR, B**

- Child Health
- Reproductive Health
- Health Systems
- Infectious Diseases and Vaccine Sciences
- HIV/AIDS
- Nutrition
- Population Sciences
- Poverty and Health

There are likely other vulnerable groups whose needs are not being met. These may include, for example, adolescents who tend to be healthier and not to use health services. Still, we know that Bangladeshi youths are malnourished, and they need sexual health information and services, and we also know that this time of life is a time when preventive strategies can reduce future health risks. The elderly may be another ‘neglected group’ in Bangladesh. As the numbers of the elderly expand and as the younger members migrate to the city, an increasing number of elderly will be left, often as widows, living in the villages without their traditional support mechanisms.

In the process of assembling this volume, the reader should recall that each paper is the collective wisdom of the members of the thematic programme of ICDDR, B. These members reviewed the available literature—both from published and unpublished sources—and assembled these data in a manner to present a message. In general, the messages are consistent across the contents. If the reader identifies a few inconsistencies in data used in one paper and that used in another, I trust this will not detract from the overall messages and the need to continue to work towards achieving the MDGs in Bangladesh.

While the information included in this volume was especially intended for USAID as it plans for future programme activities in Bangladesh, I trust it will also be useful to others who are involved with improving the health of people in Bangladesh. For persons whose work is not primarily in the public-health field, this volume may also be a valuable resource to understand the thinking and strategies for improving health and facilitating development in Bangladesh.

I want to thank all members of the programmes for their contributions, especially those forming the writing committees who were very perceptive in their ability to identify the key issues and to find the supporting data which enlighten these issues. I especially wish to thank the USAID for the financial support which has allowed our scientists the opportunity to assemble this report. This has been a productive experience for each of the programmes and will help the Centre focus its agenda on the key issues for the country during the coming years.

